# (*E*,*E*)-1,2-Bis[3-meth­oxy-4-(prop-2-yn-1-yl­oxy)benzyl­idene]hydrazine

**DOI:** 10.1107/S1600536811022410

**Published:** 2011-06-18

**Authors:** Wisam Naji Atiyah Al-Mehana, Raied M. Shakir, Rosiyah Yahya, Siti Nadiah Abd Halim, Edward R. T. Tiekink

**Affiliations:** a Department of Chemistry, University of Malaya, 50603 Kuala Lumpur, Malaysia

## Abstract

The complete mol­ecule in the title compound, C_22_H_20_N_2_O_4_, is generated by the application of an inversion centre. With the exception of the terminal acetyl­ene groups [C—O—C—C = −78.02 (17)°], the remaining atoms constituting the mol­ecule are essentially coplanar. The configuration around the C=N bond [1.282 (2) Å] is *E*. The formation of supra­molecular chains mediated by C—H⋯O inter­actions, occurring between methyl­ene H and meth­oxy O atoms, is the most notable feature of the crystal packing.

## Related literature

For background to the study see: Xu *et al.* (1997 ▶); Zheng *et al.* (2005 ▶); Kundu *et al.* (2005 ▶). For additional analysis, see: Spek (2009 ▶).
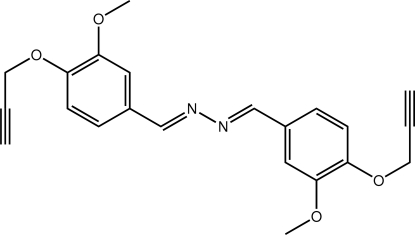


## Experimental

### 

#### Crystal data

C_22_H_20_N_2_O_4_
*M* *_r_* = 376.40
Monoclinic, 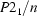

*a* = 4.4840 (3) Å
*b* = 14.4636 (8) Å
*c* = 14.3939 (9) Å
β = 91.674 (4)°
*V* = 933.11 (10) Å^3^
*Z* = 2
Mo *K*α radiation
μ = 0.09 mm^−1^
*T* = 100 K
0.25 × 0.11 × 0.07 mm

#### Data collection

Bruker SMART APEX CCD diffractometer
Absorption correction: multi-scan (*SADABS*; Sheldrick, 1996 ▶) *T*_min_ = 0.368, *T*_max_ = 0.746
8574 measured reflections
2138 independent reflections
1625 reflections with *I* > 2σ(*I*)
*R*_int_ = 0.069

#### Refinement

*R*[*F* ^2^ > 2σ(*F* ^2^)] = 0.046
*wR*(*F* ^2^) = 0.124
*S* = 1.05
2138 reflections
128 parameters
H-atom parameters constrained
Δρ_max_ = 0.26 e Å^−3^
Δρ_min_ = −0.25 e Å^−3^

### 

Data collection: *APEX2* (Bruker, 2009 ▶); cell refinement: *SAINT* (Bruker, 2009 ▶); data reduction: *SAINT*; program(s) used to solve structure: *SHELXS97* (Sheldrick, 2008 ▶); program(s) used to refine structure: *SHELXL97* (Sheldrick, 2008 ▶); molecular graphics: *ORTEP-3* (Farrugia, 1997 ▶) and *DIAMOND* (Brandenburg, 2006 ▶); software used to prepare material for publication: *publCIF* (Westrip, 2010 ▶).

## Supplementary Material

Crystal structure: contains datablock(s) global, I. DOI: 10.1107/S1600536811022410/hb5907sup1.cif

Structure factors: contains datablock(s) I. DOI: 10.1107/S1600536811022410/hb5907Isup2.hkl

Supplementary material file. DOI: 10.1107/S1600536811022410/hb5907Isup3.cml

Additional supplementary materials:  crystallographic information; 3D view; checkCIF report

## Figures and Tables

**Table 1:** Hydrogen-bond geometry (Å, °)

*D*—H⋯*A*	*D*—H	H⋯*A*	*D*⋯*A*	*D*—H⋯*A*
C8—H8a⋯O2^i^	0.99	2.36	3.255 (2)	150

Symmetry code: (i) 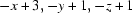.

**Fig. 1.**
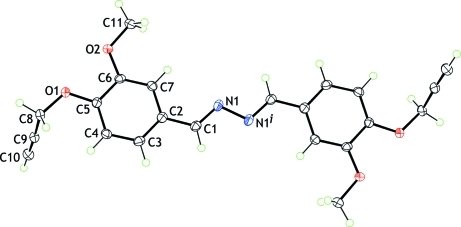
The molecular structure of centrosymmetric compound (I) showing displacement ellipsoids at the 50% probability level. Symmetry code i: 1 - x, 1 - y, -z.

**Fig. 2.**
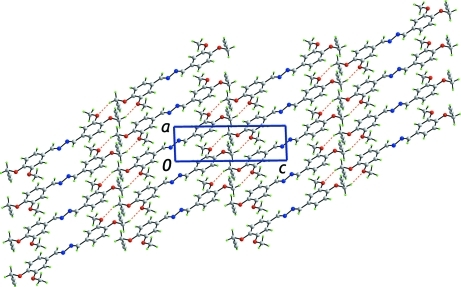
A view of the supramolecular chains in (I) mediated by C—H···O interactions (orange dashed lines) and their aggregation into layers in the ac plane.

**Fig. 3.**
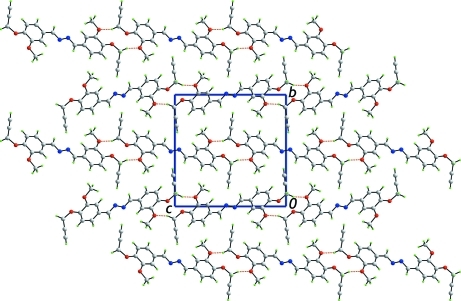
A view in projection down the a axis of the unit-cell contents for (I); the C—H···O interactions are shown as orange dashed lines.

## supplementary crystallographic information

### Comment

Molecules combining an azine functionality and/or a diimine linkage have been investigated in terms of their crystallography and coordination chemistry (Xu *et al.*, 1997; Zheng *et al.*, 2005; Kundu *et al.*, 2005). In this connection the title compound, (I), was studied.

The molecule of (I), Fig. 1, is centrosymmetric around the central azine [N1—N1^i^ = 1.413 (2) Å] bond; symmetry operation *i*: 1 - *x*, 1 - *y*, -*z*. The configuration around the C1═N1 bond [1.282 (2) Å] is *E*. With the exception of the terminal acetylene group, the molecule is essentially planar as seen in the values of the N1—C1—C2—C7 and C11—O2—C6—C5 torsion angles of 2.9 (2) and 177.04 (14) °, respectively. By contrast, the torsion angle C5—O1—C8—C9 of -78.02 (17) ° indicates the acetylene group is almost perpendicular to the rest of the molecule.

The most prominent feature of the crystal packing is the presence of C—H···O interactions, occurring between methylene-H and the methoxy-O atoms, which serve to link molecules into supramolecular chains mediated by centrosymmetric 12-membered {···HCOC_2_O}_2_ synthons, Table 1 and Fig. 2. Chains pack in the *ac* plane and interdigitate along the *b* axis., Fig. 3. Each acetylene-H atom is orientated towards an imino-N atom, being separated by 2.77 Å, *i.e*. outside the standard criteria to be considered significant (Spek, 2009).

### Experimental

Vanillinazine (2.0 g, 6.7 mmol) in dry acetone and anhydrous K_2_CO_3_ (1.84 g, 13.3 mmol) was stirred at room temperature for about 20 min. Then, an excess of propargyl bromide (1.74 g, 14.7 mmol) was added drop wise. The mixture was refluxed for 48 h. The solvent was evaporated under reduced pressure and the product extracted with 100 ml diethyl ether. The organic layer was washed with brine and dried over MgSO_4_. The yellow compound was recrystallized from ethyl acetate/methanol (1/1) solution to yield yellow needles of (I); yield 72% and *M*.pt. 460 K.

### Refinement

Carbon-bound H-atoms were placed in calculated positions (C—H 0.95 to 0.99 Å) and were included in the refinement in the riding model approximation with *U_iso_*(H) = 1.2–1.5*U_eq_*(C).

### Crystal data

**Table:**

C_22_H_20_N_2_O_4_	*F*(000) = 396
*M**_r_* = 376.40	*D*_x_ = 1.340 Mg m^−^^3^
Monoclinic, *P*2_1_/*n*	Mo *K*α radiation, λ = 0.71073 Å
Hall symbol: -P 2yn	Cell parameters from 1658 reflections
*a* = 4.4840 (3) Å	θ = 2.8–29.5°
*b* = 14.4636 (8) Å	µ = 0.09 mm^−^^1^
*c* = 14.3939 (9) Å	*T* = 100 K
β = 91.674 (4)°	Needle, yellow
*V* = 933.11 (10) Å^3^	0.25 × 0.11 × 0.07 mm
*Z* = 2	

### Data collection

**Table:**

Bruker SMART APEX CCD diffractometer	2138 independent reflections
Radiation source: fine-focus sealed tube	1625 reflections with *I* > 2σ(*I*)
graphite	*R*_int_ = 0.069
ω scans	θ_max_ = 27.5°, θ_min_ = 2.0°
Absorption correction: multi-scan (*SADABS*; Sheldrick, 1996)	*h* = −5→5
*T*_min_ = 0.368, *T*_max_ = 0.746	*k* = −18→18
8574 measured reflections	*l* = −18→18

### Refinement

**Table:**

Refinement on *F*^2^	Primary atom site location: structure-invariant direct methods
Least-squares matrix: full	Secondary atom site location: difference Fourier map
*R*[*F*^2^ > 2σ(*F*^2^)] = 0.046	Hydrogen site location: inferred from neighbouring sites
*wR*(*F*^2^) = 0.124	H-atom parameters constrained
*S* = 1.05	*w* = 1/[σ^2^(*F*_o_^2^) + (0.0481*P*)^2^ + 0.2035*P*] where *P* = (*F*_o_^2^ + 2*F*_c_^2^)/3
2138 reflections	(Δ/σ)_max_ < 0.001
128 parameters	Δρ_max_ = 0.26 e Å^−^^3^
0 restraints	Δρ_min_ = −0.25 e Å^−^^3^

### Special details

**Table:**

Geometry. All s.u.'s (except the s.u. in the dihedral angle between two l.s. planes) are estimated using the full covariance matrix. The cell s.u.'s are taken into account individually in the estimation of s.u.'s in distances, angles and torsion angles; correlations between s.u.'s in cell parameters are only used when they are defined by crystal symmetry. An approximate (isotropic) treatment of cell s.u.'s is used for estimating s.u.'s involving l.s. planes.
Refinement. Refinement of *F*^2^ against ALL reflections. The weighted *R*-factor *wR* and goodness of fit *S* are based on *F*^2^, conventional *R*-factors *R* are based on *F*, with *F* set to zero for negative *F*^2^. The threshold expression of *F*^2^ > 2σ(*F*^2^) is used only for calculating *R*-factors(gt) *etc*. and is not relevant to the choice of reflections for refinement. *R*-factors based on *F*^2^ are statistically about twice as large as those based on *F*, and *R*- factors based on ALL data will be even larger.

### Fractional atomic coordinates and isotropic or equivalent isotropic displacement parameters (Å^2^)

**Table:**

	*x*	*y*	*z*	*U*_iso_*/*U*_eq_	
O1	1.2641 (2)	0.54779 (7)	0.43315 (7)	0.0201 (3)	
O2	1.3374 (2)	0.41802 (7)	0.31509 (8)	0.0218 (3)	
N1	0.5998 (3)	0.49139 (8)	0.03789 (9)	0.0204 (3)	
C1	0.5930 (3)	0.55648 (10)	0.09829 (11)	0.0195 (4)	
H1	0.4638	0.6075	0.0867	0.023*	
C2	0.7742 (3)	0.55572 (10)	0.18375 (11)	0.0187 (3)	
C3	0.7351 (3)	0.62536 (10)	0.24843 (11)	0.0201 (4)	
H3	0.5955	0.6733	0.2351	0.024*	
C4	0.8968 (3)	0.62633 (10)	0.33254 (11)	0.0192 (3)	
H4	0.8694	0.6750	0.3758	0.023*	
C5	1.0972 (3)	0.55612 (9)	0.35270 (10)	0.0170 (3)	
C6	1.1383 (3)	0.48469 (9)	0.28727 (11)	0.0178 (3)	
C7	0.9810 (3)	0.48486 (9)	0.20414 (11)	0.0182 (3)	
H7	1.0117	0.4370	0.1602	0.022*	
C8	1.2080 (4)	0.61172 (10)	0.50702 (11)	0.0209 (4)	
H8A	1.2888	0.5857	0.5662	0.025*	
H8B	0.9898	0.6189	0.5128	0.025*	
C9	1.3423 (4)	0.70363 (10)	0.49229 (11)	0.0214 (4)	
C10	1.4542 (4)	0.77718 (11)	0.48483 (12)	0.0262 (4)	
H10	1.5441	0.8363	0.4788	0.031*	
C11	1.3762 (4)	0.34187 (10)	0.25333 (12)	0.0255 (4)	
H11A	1.1830	0.3121	0.2405	0.038*	
H11B	1.5142	0.2971	0.2821	0.038*	
H11C	1.4578	0.3642	0.1950	0.038*	

### Atomic displacement parameters (Å^2^)

**Table:**

	*U*^11^	*U*^22^	*U*^33^	*U*^12^	*U*^13^	*U*^23^
O1	0.0245 (6)	0.0180 (5)	0.0174 (6)	0.0022 (4)	−0.0053 (5)	−0.0022 (4)
O2	0.0261 (6)	0.0161 (5)	0.0228 (6)	0.0055 (4)	−0.0061 (5)	−0.0030 (4)
N1	0.0202 (7)	0.0225 (6)	0.0182 (7)	−0.0017 (5)	−0.0047 (6)	0.0031 (5)
C1	0.0185 (8)	0.0176 (7)	0.0223 (8)	−0.0017 (6)	−0.0020 (7)	0.0035 (6)
C2	0.0180 (8)	0.0171 (7)	0.0209 (8)	−0.0036 (6)	−0.0014 (7)	0.0025 (6)
C3	0.0184 (8)	0.0160 (7)	0.0260 (9)	0.0001 (6)	−0.0019 (7)	0.0029 (6)
C4	0.0221 (8)	0.0150 (7)	0.0207 (8)	−0.0009 (6)	0.0004 (7)	−0.0012 (6)
C5	0.0176 (8)	0.0162 (7)	0.0171 (8)	−0.0029 (6)	−0.0013 (6)	0.0016 (5)
C6	0.0172 (8)	0.0132 (7)	0.0230 (8)	−0.0006 (6)	−0.0007 (6)	0.0023 (6)
C7	0.0195 (8)	0.0148 (7)	0.0202 (8)	−0.0019 (6)	−0.0002 (6)	−0.0013 (5)
C8	0.0259 (9)	0.0211 (7)	0.0154 (8)	0.0006 (6)	−0.0024 (7)	−0.0026 (6)
C9	0.0245 (9)	0.0227 (8)	0.0169 (8)	0.0031 (6)	−0.0020 (7)	−0.0032 (6)
C10	0.0325 (10)	0.0215 (8)	0.0244 (9)	0.0001 (7)	−0.0031 (8)	−0.0031 (6)
C11	0.0293 (9)	0.0176 (7)	0.0293 (9)	0.0034 (6)	−0.0056 (8)	−0.0055 (6)

### Geometric parameters (Å, °)

**Table:**

O1—C5	1.3655 (17)	C4—H4	0.9500
O1—C8	1.4368 (18)	C5—C6	1.414 (2)
O2—C6	1.3664 (16)	C6—C7	1.371 (2)
O2—C11	1.4292 (18)	C7—H7	0.9500
N1—C1	1.282 (2)	C8—C9	1.477 (2)
N1—N1^i^	1.413 (2)	C8—H8A	0.9900
C1—C2	1.454 (2)	C8—H8B	0.9900
C1—H1	0.9500	C9—C10	1.182 (2)
C2—C3	1.386 (2)	C10—H10	0.9500
C2—C7	1.407 (2)	C11—H11A	0.9800
C3—C4	1.393 (2)	C11—H11B	0.9800
C3—H3	0.9500	C11—H11C	0.9800
C4—C5	1.381 (2)		
			
C5—O1—C8	117.87 (11)	O2—C6—C5	114.66 (13)
C6—O2—C11	116.87 (11)	C7—C6—C5	120.43 (13)
C1—N1—N1^i^	111.53 (15)	C6—C7—C2	120.15 (14)
N1—C1—C2	122.96 (14)	C6—C7—H7	119.9
N1—C1—H1	118.5	C2—C7—H7	119.9
C2—C1—H1	118.5	O1—C8—C9	113.08 (13)
C3—C2—C7	118.98 (14)	O1—C8—H8A	109.0
C3—C2—C1	118.95 (13)	C9—C8—H8A	109.0
C7—C2—C1	122.03 (14)	O1—C8—H8B	109.0
C2—C3—C4	121.22 (14)	C9—C8—H8B	109.0
C2—C3—H3	119.4	H8A—C8—H8B	107.8
C4—C3—H3	119.4	C10—C9—C8	176.86 (17)
C5—C4—C3	119.61 (14)	C9—C10—H10	180.0
C5—C4—H4	120.2	O2—C11—H11A	109.5
C3—C4—H4	120.2	O2—C11—H11B	109.5
O1—C5—C4	125.46 (13)	H11A—C11—H11B	109.5
O1—C5—C6	114.94 (12)	O2—C11—H11C	109.5
C4—C5—C6	119.60 (13)	H11A—C11—H11C	109.5
O2—C6—C7	124.90 (13)	H11B—C11—H11C	109.5
			
N1^i^—N1—C1—C2	179.28 (15)	C11—O2—C6—C5	177.04 (14)
N1—C1—C2—C3	−174.95 (15)	O1—C5—C6—O2	0.4 (2)
N1—C1—C2—C7	2.9 (2)	C4—C5—C6—O2	−178.85 (13)
C7—C2—C3—C4	0.2 (2)	O1—C5—C6—C7	179.38 (13)
C1—C2—C3—C4	178.11 (14)	C4—C5—C6—C7	0.1 (2)
C2—C3—C4—C5	−0.9 (2)	O2—C6—C7—C2	178.03 (14)
C8—O1—C5—C4	6.4 (2)	C5—C6—C7—C2	−0.8 (2)
C8—O1—C5—C6	−172.80 (13)	C3—C2—C7—C6	0.7 (2)
C3—C4—C5—O1	−178.48 (15)	C1—C2—C7—C6	−177.22 (15)
C3—C4—C5—C6	0.7 (2)	C5—O1—C8—C9	−78.02 (17)
C11—O2—C6—C7	−1.8 (2)	O1—C8—C9—C10	−136 (3)

Symmetry codes: (i) −*x*+1, −*y*+1, −*z*.

### Hydrogen-bond geometry (Å, °)

**Table:**

*D*—H···*A*	*D*—H	H···*A*	*D*···*A*	*D*—H···*A*
C8—H8a···O2^ii^	0.99	2.36	3.255 (2)	150

Symmetry codes: (ii) −*x*+3, −*y*+1, −*z*+1.
